# Extension in Hip Disease

**Published:** 1870-11

**Authors:** 


### Miscellaneous.

### Extension in Hip Disease.

Dr. Clinton Cushing, of California, concludes an account of two cases of hip disease, as follows: 1. Perfect rest, as near as possible, is of the first importance in the treatment of hip disease, at whatever stage. 2. The best way to secure this is by moderate extension, by means of a weight and pulley. 3. By this means the opposing diseased surfaces are kept asunder, and by the same means the patient is allowed to move about on the bed quite freely, without aggravating the disease of the joint. It is surprising to see how well children will bear confinement to a bed in the manner indicated, and how readily they accept the situation when they are free from pain. He thinks that a tonic course of treatment is demanded in every case, at whatever stage; and is a firm believer in the virtues of counter-irritation in this disease, thoroughly applied.†—*Pacific Medical and Surgical Journal.*

† A thorough *disbeliever*, and if vigorously applied, *barbarous*. [Ed. Buffalo Med. Journal.

